# Career Plateau Among Surgical Nurses: A Cross‐Sectional Network Analysis

**DOI:** 10.1155/jonm/6676263

**Published:** 2026-07-12

**Authors:** Duo Zhang, Fan Yang, Haoyang Zheng, Yanrong Zhou, Juan Liu

**Affiliations:** ^1^ Department of Nursing, Tongji Hospital, Tongji Medical College, Huazhong University of Science and Technology, Wuhan, Hubei, China, hust.edu.cn; ^2^ Department of Neurosurgery, The First Medical Center, Chinese PLA General Hospital, Beijing, China, 301hospital.com.cn

**Keywords:** career development, career plateau, network analysis, nurses

## Abstract

**Background:**

Career plateau is increasingly recognized as a threat to nursing workforce stability because it is associated with poorer work performance, lower organizational commitment, and higher turnover intention. Surgical nurses may be especially vulnerable given their highly structured and technically demanding work context. However, evidence remains limited on how career plateau is configured in this group and how relevant factors are interrelated.

**Aim:**

Based on Bandura’s triadic reciprocal determinism, this study examined career plateau among surgical nurses and its relationships with self‐efficacy as an individual factor, family functioning and perceived organizational support as environmental factors, and career planning as a behavioral factor.

**Methods:**

A cross‐sectional study was conducted among 468 surgical nurses from three hospitals in Hubei Province, China, between September and November 2025. Data were collected through an online survey. Dominance analysis was used to compare the relative contribution of study variables to overall career plateau, and psychometric network analysis was used to examine subdimension‐level relationships.

**Results:**

Career planning was negatively associated with career plateau and showed the largest relative contribution to overall career plateau. In the network, affection emerged as the most central node. Strong negative associations were observed between hierarchical plateau and goal setting and between content plateau and self‐presentation, whereas emotional support and instrumental organizational support were strongly positively associated.

**Conclusion:**

Career planning appears to be the most important broad correlate of overall career plateau, whereas affection occupies a central position within the network structure. Reducing career plateau in surgical nurses may therefore require not only stronger career planning but also family and organizational conditions that make such planning realistic and sustainable.

**Implications for Nursing Management:**

Nurse managers should prioritize structured career planning for surgical nurses and foster family‐sensitive organizational conditions that make career development more feasible and sustainable. Particular attention should be given to mentoring, realistic advancement opportunities, and work arrangements that reduce conflict between career goals and family demands.

## 1. Introduction

Career plateau refers to a state in which employees face limited opportunities for further advancement or role expansion in their careers [1]. It is commonly conceptualized as a multidimensional construct comprising hierarchical plateau, content plateau, and centralization plateau, which denote restricted promotion prospects, reduced challenge in work content, and limited involvement in core organizational roles, respectively [2]. When accumulated expertise is not matched by opportunities for growth, career plateau may emerge and has been associated with lower job satisfaction, greater burnout, and stronger turnover intention among healthcare professionals [1–3]. Such consequences threaten workforce stability and may ultimately undermine the sustainability and effectiveness of healthcare systems.

Nurses are central to patient care, health management, and service delivery, yet career advancement in nursing often remains constrained by rigid role hierarchies, limited skill‐development pathways, and insufficient organizational support for expanded roles [2, 4, 5]. Previous studies suggest that approximately 34% of nurses experience career plateau, indicating that professional stagnation is not uncommon [5]. However, this evidence has been derived largely from general nursing samples, leaving career plateau in surgical nurses insufficiently characterized.

Surgical nursing is characterized by standardized procedures, high technical demands, and relatively stable role structures that require sustained performance in fast‐paced and high‐risk settings [6]. Although such conditions foster technical mastery, they may also constrain both vertical mobility, such as promotion, and horizontal mobility, such as expansion into education, research, management, or clinical leadership [7, 8]. Opportunities to apply advanced competencies may therefore remain limited, even among nurses with substantial expertise. This imbalance between high professional demand and restricted developmental opportunity may reduce professional fulfillment, weaken motivation for advancement, and increase vulnerability to career plateau in surgical settings [3]. Qualitative evidence also suggests that nurses experiencing career plateau may report blocked development, negative emotions, and weakened professional commitment, underscoring the need to examine career plateau as a phenomenon embedded in broader structural and psychosocial conditions [2].

Bandura’s triadic reciprocal determinism provides a useful framework for understanding this phenomenon [9]. The model emphasizes the dynamic interplay of personal factors, environmental conditions, and behavior in shaping career‐related outcomes. Within this framework, self‐efficacy was conceptualized as the personal component, family functioning and perceived organizational support as environmental components, and career planning as the behavioral component. Existing evidence indicates that age and educational background may shape nurses’ career trajectories by influencing promotion opportunities, role mobility, and access to development [5, 10, 11]. At the same time, insufficient organizational support may intensify career plateau by limiting recognition, resources, and developmental opportunities, whereas self‐efficacy and family functioning may help sustain adaptive engagement with career challenges under demanding work conditions [5, 12].

Despite these insights, previous studies have relied predominantly on regression‐based analyses or structural equation modeling [1, 3, 5]. Although these approaches can identify specific pathways and indirect effects, they usually depend on prespecified directional assumptions and are less suited to depicting patterns of interdependence among multiple factors. Career plateau is therefore unlikely to be adequately explained by any single predictor and is more plausibly shaped by the joint configuration of personal resources, organizational conditions, social support, and career‐related behaviors [5, 10–12].

Network analysis conceptualizes variables as nodes and their associations as edges, offering a system‐level representation of complex phenomena. In addition to identifying direct relationships, it can identify comparatively central components within a broader structure of interdependence [13]. This approach has been increasingly applied in health research to examine interrelated psychosocial and occupational variables [13, 14]. Guided by Bandura’s triadic reciprocal determinism, this study aimed to examine the network structure of personal, environmental, and behavioral factors associated with career plateau among surgical nurses. Specifically, the study addressed three questions: What direct relationships exist among career plateau, self‐efficacy, family functioning, perceived organizational support, and career planning; which factors occupy the most central positions within the network; and which nodes may represent priority targets for future intervention development.

## 2. Materials and Methods

### 2.1. Design

This study employed a cross‐sectional design.

### 2.2. Theoretical Framework

**FIGURE 1:**
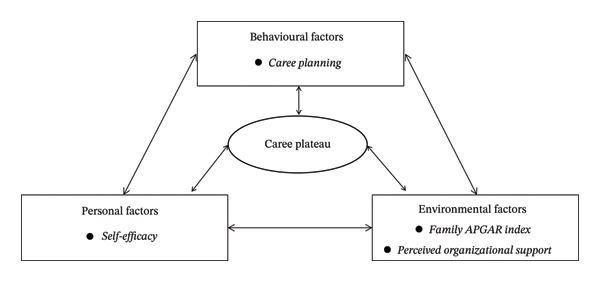
Hypothesized relationships among the study variables.

Triadic reciprocal determinism provided the theoretical framework for this study. Family functioning and perceived organizational support were considered external resources that may alleviate work‐related stress and facilitate work engagement, whereas self‐efficacy and career planning reflected nurses’ confidence and active efforts in managing career development. Career plateau was therefore conceptualized as a condition arising from the interplay of personal, behavioral, and environmental factors. As a theoretical assumption within this framework, career plateau may be linked to reduced self‐efficacy, less active career planning, and less effective use of available support (Figure 1).

### 2.3. Sample

This cross‐sectional study was conducted between September and November 2025 in three large tertiary Grade A public hospitals in Wuhan, Hubei Province, China. A total of 486 nurses were recruited using convenience sampling. Eligibility criteria were as follows: (1) holding a valid and registered nursing license; (2) having at least 3 years of experience working in surgical departments; and (3) providing informed consent and participating voluntarily. Nurses who were on leave during the survey period were excluded.

Sample size was estimated using the cross‐sectional formula $N={\left(Z_{\alpha /2}\cdot \sigma /\delta \right)}^{2}$, with *Z*_*α*/2_ = 1.96. The standard deviation (SD) (*σ*) was set at 0.99 based on prior research by Qiao [15], and the allowable error (*δ*) was set at 0.10 to ensure adequate estimation precision. After accounting for a 10% nonresponse rate, the minimum required sample size was calculated to be 419.

### 2.4. Measures

#### 2.4.1. General Information Questionnaire

A researcher‐developed questionnaire was used to collect demographic and professional characteristics, including age, gender, and professional title.

#### 2.4.2. Family APGAR Questionnaire

The Family APGAR Questionnaire was developed by Smilkstein [16] and translated into Chinese by Lv et al. [17]. The scale comprises five items assessing adaptation, partnership, growth, affection, and resolve. Each item is scored on a scale from 0 to 2, yielding a total score ranging from 0 to 10, with higher scores indicating greater satisfaction with family functioning. The scale Cronbach’s alpha was 0.896.

#### 2.4.3. General Self‐Efficacy Scale

Self‐efficacy was measured using a three‐item shortened scale developed by Yu et al., based on the original instrument by Chen et al. [18, 19]. The items assess individuals’ perceived confidence and coping ability when facing tasks and challenges and are rated on a five‐point Likert scale ranging from 1 (*strongly disagree*) to 5 (*strongly agree*). Higher scores indicate higher self‐efficacy. The scale Cronbach’s alpha was 0.838.

#### 2.4.4. Perceived Organizational Support Scale

Perceived organizational support was assessed using a 13‐item scale [20]. The scale comprises two dimensions: emotional support and instrumental support. Items are rated on a five‐point Likert scale from 1 (*very inconsistent*) to 5 (*very consistent*), with higher scores indicating stronger perceived organizational support. The scale Cronbach’s alpha was 0.921.

#### 2.4.5. Career Planning Questionnaire

The scale was used to assess nurses’ career planning level [21, 22]. It includes 14 items across four dimensions: goal setting, continuous learning, self‐presentation, and relationship building. Items are rated on a four‐point Likert scale from 1 (*inconsistent*) to 4 (*consistent*), yielding total scores ranging from 14 to 56. Higher scores indicate better career planning. The scale Cronbach’s alpha was 0.820.

#### 2.4.6. Career Plateau Scale

Career plateau was assessed using a 16‐item scale comprising three dimensions: hierarchical plateau, content plateau, and centralization plateau, which evaluate perceived limitations in promotion, job content, and role centrality, respectively [23]. Items are rated on a six‐point Likert scale ranging from 1 (*strongly disagree*) to 6 (*strongly agree*). Dimension scores are calculated as the mean of item scores, and the overall score is the mean of the three‐dimension scores. Scores above 3 indicate the presence of career plateau, with higher scores reflecting greater severity. The scale Cronbach’s alpha was 0.821.

### 2.5. Data Collection

Data were collected using an online questionnaire administered via the Wenjuanxing platform. A pilot survey was conducted prior to formal distribution to assess logical coherence and usability, and the questionnaire was refined based on participant feedback. Participation was voluntary, and informed consent was obtained from all respondents. Data quality was ensured through mandatory response settings, logic checks, and restrictions on multiple submissions from the same IP address. Data cleaning and entry were independently performed by two researchers. Questionnaires with completion times of less than five minutes or with highly uniform response patterns were excluded; the time threshold was determined based on pilot testing to identify potentially inattentive responses. Regular team meetings were held throughout data collection to monitor progress and reinforce quality control procedures.

### 2.6. Data Analysis

All analyses were conducted using R Version 4.5.1. Continuous variables are reported as means and SDs, and categorical variables as frequencies and percentages. Pearson correlation coefficients were calculated to examine associations among study variables.

For correlation and dominance analyses, career plateau was represented by the overall score as an index of overall plateau level, and dominance analysis was used to assess the relative importance of family functioning, self‐efficacy, perceived organizational support, and career planning in predicting career plateau across all possible subset regression models [24]. In the network analysis, hierarchical plateau, content plateau, and centralization plateau were modeled as separate nodes to capture subdimension‐level associations.

Fifteen variables were specified as network nodes. Nodes were connected by edges representing associations, with edge thickness indicating association strength and edge color indicating direction (red for positive and blue for negative). Network estimation and visualization were performed using the R packages qgraph and bootnet [25, 26]. The network was estimated using the graphical least absolute shrinkage and selection operator (gLASSO) with the extended Bayesian information criterion (EBIC), with the tuning parameter *γ* set to 0.5 [27].

Node importance was assessed using four centrality indices: strength, closeness, betweenness, and expected influence (EI) [28]. Strength reflects the sum of edge weights connected to a node, closeness indicates the average distance to all other nodes, and betweenness represents the frequency with which a node lies on shortest paths between other nodes. EI extends strength by accounting for the direction of associations, capturing a node’s potential positive or negative impact on the network. All centrality indices were standardized as *z* scores.

Network accuracy and stability were evaluated using bootnet. Nonparametric bootstrap procedures were applied to estimate confidence intervals for edge weights based on 1500 resamples, with narrower intervals indicating greater estimation precision [25]. Bootstrap difference tests were used to assess significant differences in network properties. Centrality stability was examined using the correlation stability (CS) coefficient, which reflects the maximum proportion of cases that can be removed while maintaining a correlation of at least 0.70 with the original centrality estimates [29]. CS values above 0.25 were considered acceptable, and values above 0.50 were considered optimal [25].

### 2.7. Ethical Considerations

The study was approved by the Ethics Committee of Tongji Hospital, Tongji Medical College, Huazhong University of Science and Technology (TJ‐IRB202406051). Before accessing the electronic questionnaire, all eligible participants were provided with information about the study purpose, procedures, voluntary nature of participation, confidentiality measures, and their right to withdraw at any time without any adverse consequences. Electronic informed consent was obtained from all participants before questionnaire completion. Participant privacy and data security were strictly protected, with all data anonymized and accessible only to the research team, and used exclusively for research purposes. The study was conducted in accordance with the Declaration of Helsinki and relevant ethical guidelines.

## 3. Results

### 3.1. Sociodemographic Characteristics

A total of 486 questionnaires were distributed. After quality control, 12 questionnaires with completion times under five minutes and 6 with highly patterned responses were excluded. The final sample comprised 468 valid questionnaires, yielding a response rate of 96.30%. The mean age of participants was 34.16 years (SD 5.29), and the mean length of professional experience was 12.79 years (SD 5.40) (see Table 1).

**TABLE 1:** General characteristics of the participants (*N* = 468).

Variable	Category	*N* (%)
Gender	Male	43 (9.19)
	Female	425 (90.81)

Professional title	Junior	166 (35.47)
	Intermediate	281 (60.04)
	Senior	21 (4.49)

Educational level	Bachelor’s degree	403 (86.11)
	Master’s degree or above	65 (13.89)

Marital status	Married	348 (74.36)
	Unmarried or divorced	120 (25.64)

Having children	Yes	271 (57.91)
	No	197 (42.09)

Employment type	Contract based	415 (88.68)
	Permanent	53 (11.32)

Monthly income (CNY)	≤ 8999	99 (21.15)
	9000∼16,999	283 (60.47)
	≥ 17,000	86 (18.38)

Number of night shifts per month	≤ 4	155 (33.12)
	5∼8	278 (59.40)
	≥ 9	35 (7.48)

### 3.2. Descriptive Statistics and Correlation Analysis

**FIGURE 2:**
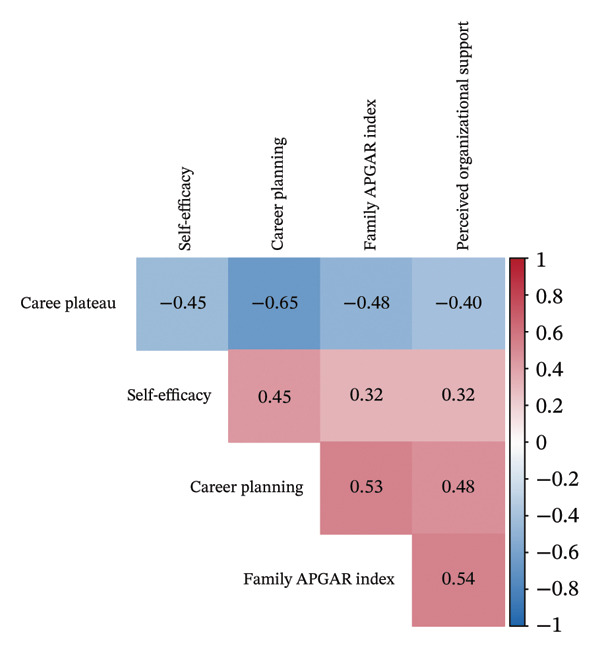
Correlations among variables.

The mean scores for the family APGAR index, self‐efficacy, perceived organizational support, career planning, and career plateau were 8.08 (SD 1.60), 11.90 (SD 1.51), 45.96 (SD 6.12), 40.72 (SD 5.35), and 3.62 (SD 0.35), respectively. All variables were significantly correlated with one another (*p* < 0.05). Detailed correlation results are presented in Figure 2, supporting the use of dominance analysis to compare the relative importance of predictors.

### 3.3. Dominance Analysis

Dominance analysis results (Table 2) showed that career planning contributed the largest share of explained variance in career plateau, followed by family APGAR index and self‐efficacy, whereas perceived organizational support contributed the smallest incremental variance.

**TABLE 2:** Dominance analysis: average *R* squared across subsets (*n* = 468).

Outcome variable	Number of predictors in the model	Family APGAR index	Self‐efficacy	Perceived organizational support	Career planning
Career plateau	0	0.230	0.202	0.163	0.424
	1	0.083	0.081	0.038	0.248
	2	0.034	0.044	0.008	0.174
	3	0.014	0.024	0.001	0.135
	General dominance	0.090	0.088	0.053	0.245

### 3.4. Network Analysis

**FIGURE 3:**
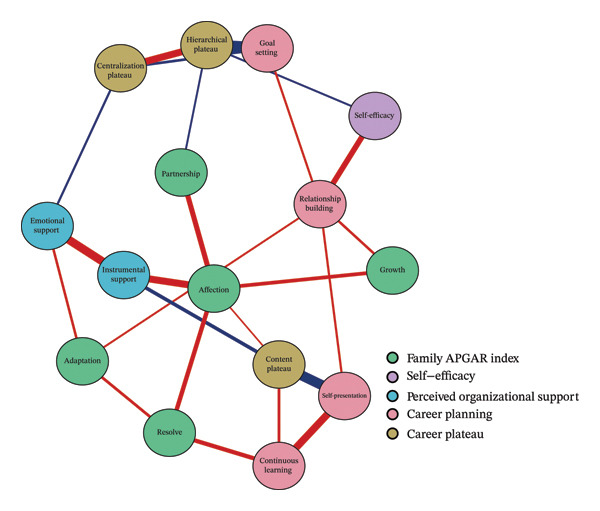
Network visualization (*n* = 468).

Figure 3 presents the estimated network model. Of the 105 possible edges, 24 showed nonzero associations, accounting for 22.86% of all potential connections. Strong positive associations were observed between emotional support and instrumental support (weight = 0.440), continuous learning and self‐presentation (weight = 0.395), and resolve and instrumental support (weight = 0.380). In contrast, strong negative associations were identified between hierarchical plateau and goal setting (weight = −0.590) and between self‐presentation and content plateau (weight = −0.516). Detailed edge weight information is provided in Supporting Figure 1, which presents a heatmap representation of edge weights between nodes.

**FIGURE 4:**
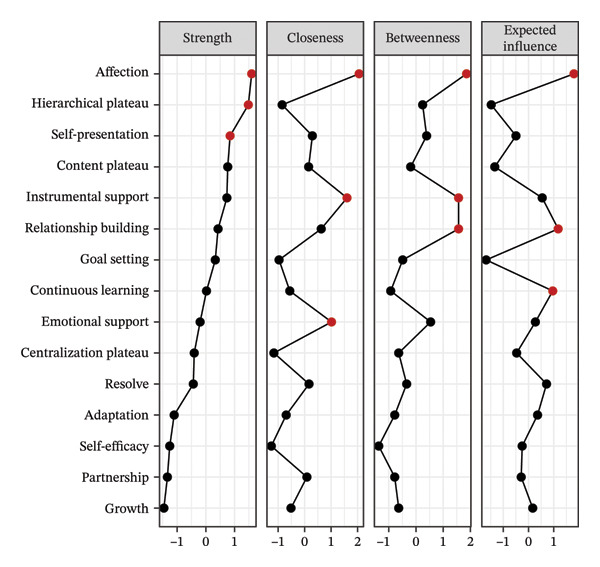
Centrality indices: strength, closeness, betweenness, and expected influence. Note. Nodes are numbered sequentially on the *y*‐axis. The *x*‐axis shows standardized centrality values (*z* scores), with higher values indicating greater centrality. Nodes highlighted in red rank relatively higher on the corresponding centrality measure.

Centrality analysis revealed distinct roles of nodes across indices. For betweenness, affection ranked highest (22), indicating a key bridging role in information flow. In closeness, affection (0.0090), instrumental support (0.0087), and emotional support (0.0082) showed the highest values, reflecting greater proximity to other nodes. Strength centrality identified affection (1.288), hierarchical plateau (1.254), and self‐presentation (1.069) as the most strongly connected nodes. In terms of EI, affection (1.288), relationship building (0.947), and continuous learning (0.830) contributed most to sustaining and spreading network effects, whereas goal setting showed a pronounced negative influence (−0.605). Centrality indices for nodes are reported in Supporting Table 1, and the corresponding key findings are summarized in Figure 4.

### 3.5. Accuracy and Stability of the Network

**FIGURE 5:**
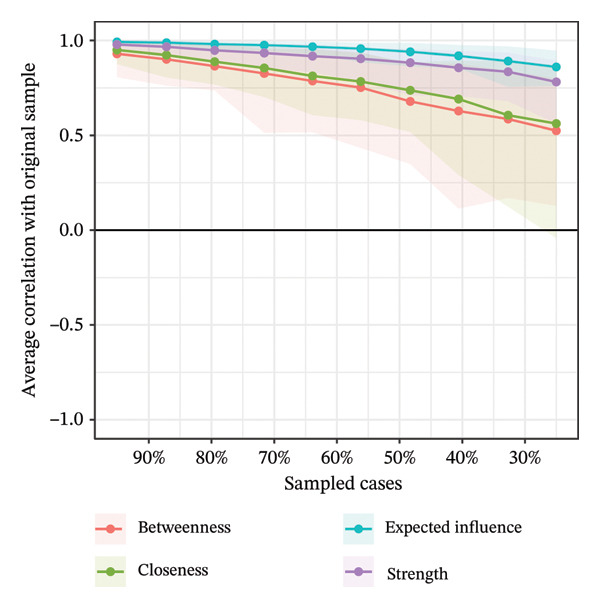
Correlation stability coefficients for strength, closeness, and betweenness. Note. The plot shows correlations between centrality indices estimated from the full sample and from subsets generated by case‐dropping bootstrap, with sample proportions decreasing to 30%.

Network accuracy was evaluated using case‐dropping bootstrap procedures. Edge weight confidence intervals were generally narrow and overlapping, indicating acceptable estimation precision. The full confidence interval results are shown in Supporting Figure 2. Robustness was adequate for strength and EI, with CS coefficients of 0.594 and 0.750, respectively; additional stability metrics are provided in Supporting Table 2, alongside the main results in Figure 5. Bootstrap difference tests for edge weights are shown in Supporting Figure 3, where black boxes indicate edge weights that differ significantly from one another. Several stronger edges differed significantly from weaker edges, supporting the relative distinctiveness of the main associations retained in the network.

## 4. Discussion

There are few studies examining career plateau among surgical nurses using network analysis. In the present study, dominance analysis and network analysis addressed two related but distinct aspects of career plateau. Dominance analysis identified the relative importance of broad correlates of the overall plateau level, whereas network analysis identified central components within the network structure. Considered together, the findings suggest that career planning was the strongest broad correlate of overall career plateau, whereas affection was the most central subdimension within the network. This distinction provides a clearer basis for interpreting the results and their implications for nursing management.

Dominance analysis showed that career planning accounted for the largest share of explained variance in career plateau. Conceptually, career planning reflects career direction, goal formulation, and the extent to which professional intentions can be translated into concrete developmental actions. In highly structured and technically intensive surgical settings, clearer planning may be particularly important because opportunities for advancement are often constrained, making individual planning more relevant for sustaining a sense of development [30, 31]. In comparison, family functioning and self‐efficacy may function as general adaptive resources, whereas perceived organizational support contributed less incremental explanatory value when modeled alongside the other predictors. These findings support greater attention to structured career planning support, including mentoring, career coaching, and individualized development plans that help nurses align professional goals with realistic opportunities for advancement.

The estimated network was relatively sparse; however, several strong associations indicate that career plateau among surgical nurses is shaped by a limited set of closely interrelated factors rather than diffuse influences [30–32]. This sparse structure, obtained with *γ* = 0.5, reflects a relatively conservative edge‐selection strategy, such that only the stronger relationships were retained in the final network [33]. The relationship between hierarchical plateau and goal setting suggests that when nurses perceive promotion pathways as blocked, they are less likely to actively engage in career goal setting. This pattern is more consistent with a rational response to limited advancement opportunities than with a lack of professional aspiration [34]. Self‐efficacy was also associated with more active relationship building and lower hierarchical plateau, suggesting that confidence in one’s capabilities may be linked to more adaptive professional engagement, although these associations should not be interpreted causally in a cross‐sectional study. The close alignment between emotional and instrumental support highlights the integrated nature of support resources, indicating that tangible assistance may be most effective when embedded in relational contexts characterized by trust, recognition, and care.

Interpretation of node importance placed greater emphasis on strength and EI, given their stronger stability in the present network [25]. Closeness and betweenness were treated as supplementary and interpreted cautiously. On this basis, affection, hierarchical plateau, and self‐presentation emerged as the most structurally prominent components. Affection may represent an important emotional resource within the family context, consistent with evidence that affective family functioning is associated with better emotional regulation and psychological well‐being among nurses [35, 36]. Hierarchical plateau may reflect perceived structural constraints in career advancement. In the Chinese context, promotion of clinical nurses is often evaluated not only on clinical performance but also on teaching and research achievements [37]. Limited access to academic resources and development opportunities may therefore be related to earlier perceptions of restricted mobility [38]. Self‐presentation may represent an active strategy through which nurses seek recognition, strengthen professional relationships, and improve access to developmental opportunities [39]. In addition, the negative EI of goal setting suggests that stronger goal‐setting tendencies were more closely linked to lower levels of constraining network features, although this pattern should be interpreted as relational rather than directional.

Additional network findings further underscored the role of organizational and interpersonal resources. Instrumental support emerged as a broadly connected component, suggesting that tangible resources may be linked to multiple domains within the network. Tangible resources, such as flexible scheduling, access to training, and career development opportunities, may facilitate the integration and diffusion of supportive effects, particularly when they are embedded within relational contexts that promote emotion regulation and psychological resilience [38, 40]. Relationship building highlighted the importance of positive interpersonal relationships and teamwork in alleviating career stagnation, consistent with evidence on the moderating role of team climate in career development [41]. Notably, the relatively large negative EI of goal setting indicates that its associations within the network are predominantly inhibitory, suggesting stronger links to constraining conditions than to activating career‐related or psychological factors. These findings do not rule out the possibility that goal setting is embedded in a context of career stagnation [39]. Interventions should therefore move beyond encouraging goal setting alone and strengthen the conditions that make goals workable, including tangible organizational support and accessible development platforms.

The practical implications of these findings are most relevant at three levels. At the unit level, nurse managers may prioritize mentoring, career coaching, individualized development planning, and more predictable scheduling arrangements, particularly for nurses who report unclear promotion prospects or diminished career direction. At the hospital level, clearer promotion criteria, recognition of clinical contributions, and better access to teaching and research resources may help address the structural barriers reflected in hierarchical plateau. At the organizational or policy level, family‐friendly practices and career development systems should be better aligned to support feasible and transparent pathways for professional growth. Together, these measures are more closely aligned with the present findings than general recommendations to increase support alone.

## 5. Limitations

Several limitations should be considered. First, the cross‐sectional design precludes causal inference and limits examination of temporal dynamics among variables; thus, the observed network structure reflects associations rather than directional effects. Second, all data were derived from self‐reported questionnaires, which may introduce common method bias, subjective distortion, and social desirability bias, although validated instruments and strict quality control procedures were applied. The online format may also have favored nurses more comfortable with digital participation, and attention control relied mainly on response time and response‐pattern screening. Third, participants were recruited from surgical departments of tertiary hospitals in a single region using convenience sampling, which may restrict the generalizability of the findings to other clinical settings or healthcare systems, particularly primary care, internal medicine, and chronic care settings. In addition, network analysis identifies structural relationships and central nodes but does not directly test underlying mechanisms, and some potentially relevant organizational or policy‐level factors were not included. Accordingly, the findings should be interpreted as exploratory, and future studies using longitudinal designs, multisource data, and broader variable frameworks are needed to further validate and extend these results.

## 6. Conclusion

This study showed that career planning made the largest relative contribution to overall career plateau, whereas affection occupied the most central position in the network. These findings suggest that reducing career plateau in surgical nurses should primarily involve strengthening career planning, particularly by improving the alignment between career goals and realistic development opportunities. At the same time, stronger family emotional conditions reflected in affection and more family‐friendly organizational policies may help make such planning more feasible and sustainable.

## Author Contributions

Duo Zhang: conceptualization, methodology, formal analysis, writing–original draft, and writing–review and editing. Fan Yang: conceptualization, methodology, investigation, data curation, and writing–original draft. Haoyang Zheng: data curation, software, formal analysis, and visualization. Yanrong Zhou: investigation, resources, and validation. Juan Liu: project administration, supervision, and writing–review and editing.

## Funding

This research received funding support from Huazhong University of Science and Technology (TJJXYJ2025070, 2025C06, 2024D14, 2024D30, and 2023C09).

## Disclosure

All authors critically reviewed the manuscript, approved the final version, and agreed to be accountable for all aspects of the work.

## Conflicts of Interest

The authors declare no conflicts of interest.

## Supporting Information

Additional supporting information can be found online in the Supporting Information section.

## Supporting information


**Supporting Information** Supporting Figure 1. Heatmap representation of edge weights between nodes. Supporting Figure 2. Bootstrap‐estimated confidence intervals for edge weights. Supporting Figure 3. Bootstrap difference tests for edge weights in the network. Supporting Table 1. Centrality indices for the network. Supporting Table 2. CS coefficients for centrality measures in the network.

## Data Availability

The data that support the findings of this study are available from the corresponding author upon reasonable request.
