# Supplementary material for: Career Plateau Among Surgical Nurses: A Cross‐Sectional Network Analysis
Source: J Nurs Manag. 2026 Jul 12;2026:6676263. doi: 10.1155/jonm/6676263 (PMC13358206; doi:10.1155/jonm/6676263)
Supplement: Supplementary file 1 — Supporting Information Supporting Figure 1. Heatmap representation of edge weights between nodes. Supporting Figure 2. Bootstrap‐estimated confidence intervals for edge weights. Supporting Figure 3. Bootstrap difference tests for edge weights in the network. Supporting Table 1. Centrality indices for the network. Supporting Table 2. CS coefficients for centrality measures in the network. [file JONM-2026-6676263-s001.pdf]

Supplementary Figure 1

Heatmap Representation of Edge Weights Between Nodes

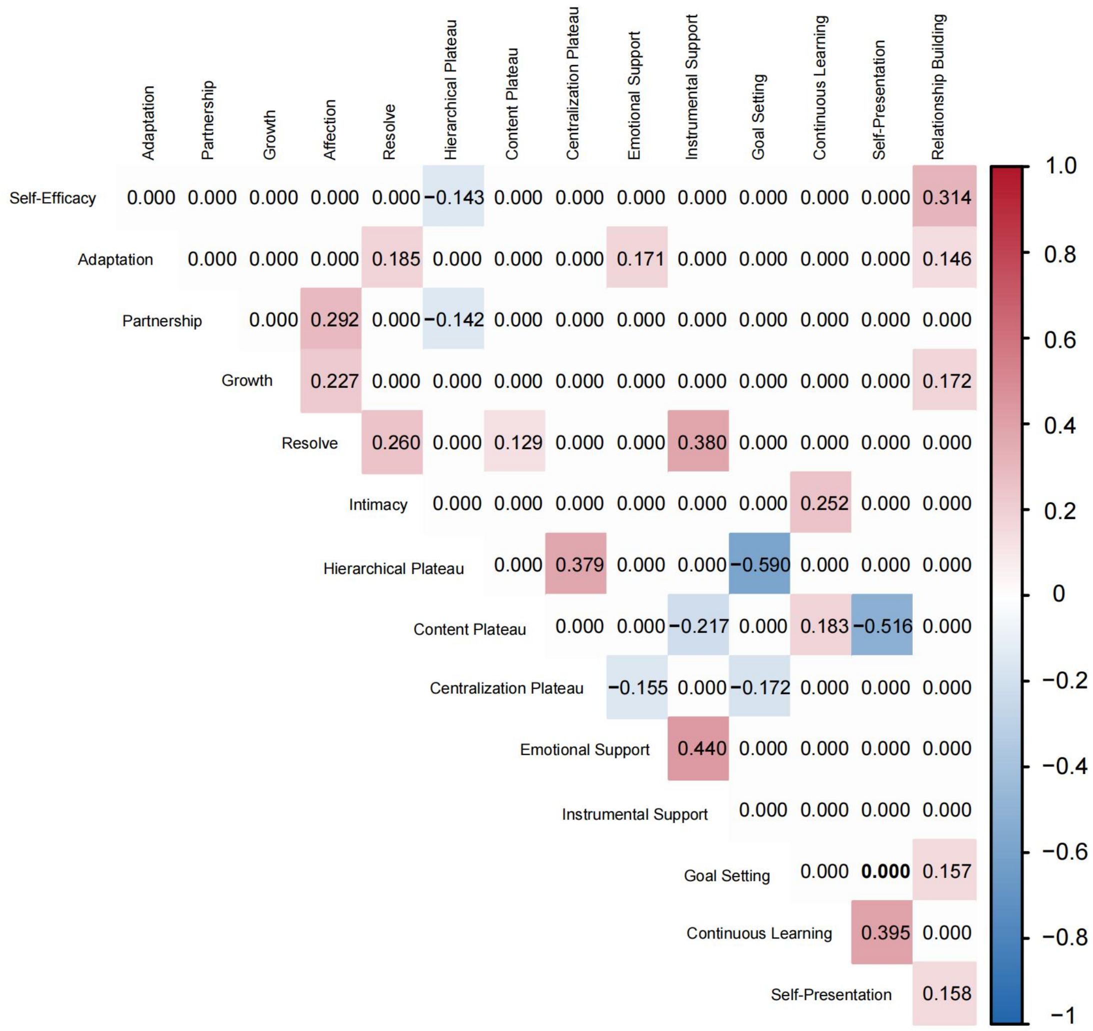

Supplementary Figure 2

Bootstrap-estimated confidence intervals for edge weights.

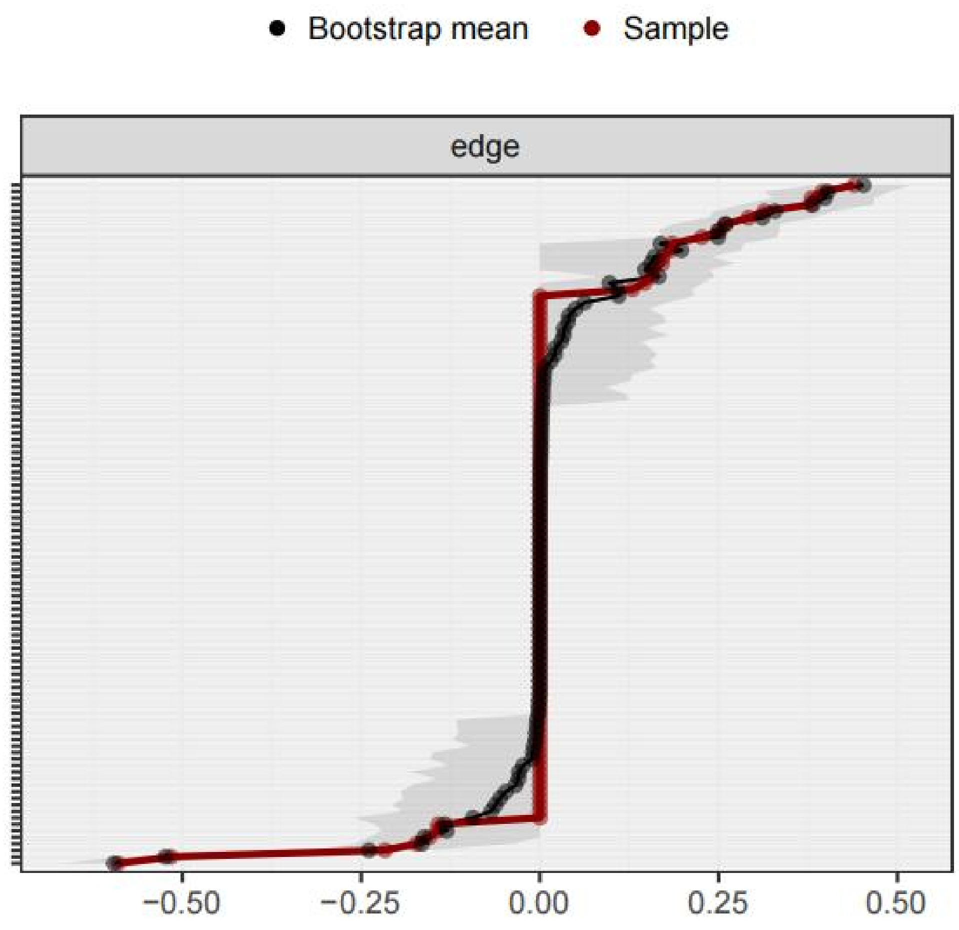

Note. The red points connected by lines represent bootstrap-estimated edge weights arranged in descending order. The black points indicate sample estimates, and the gray area represents the 95% confidence interval. Narrower intervals indicate greater precision of the edge-weight estimates.

Supplementary Figure 3

Bootstrap difference tests for edge weights in the network.

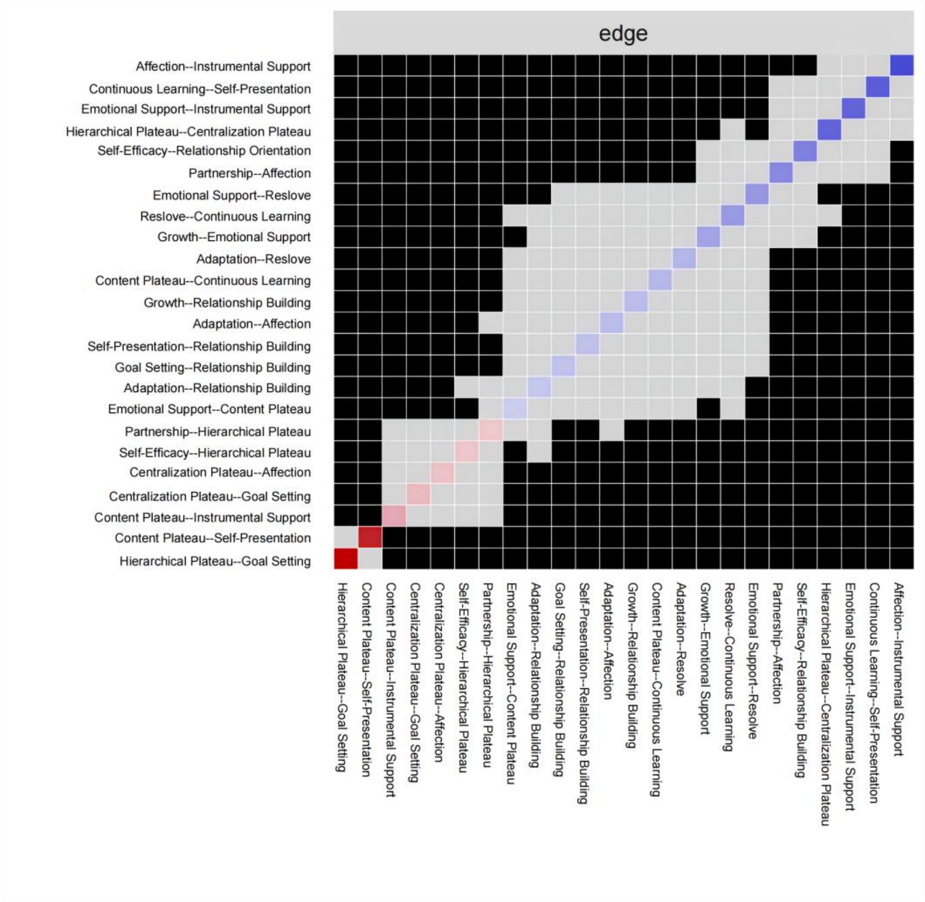

Note. Gray boxes indicate edge weights that do not differ significantly from one another, whereas black boxes indicate significant differences. Blue boxes on the diagonal denote positive edge weights.

Supplementary Table 1. Centrality Indices for the network.

Node	Betweenness	Closeness	Strength	Expected Influence
Self-Efficacy	0	0.006441795	0.4576048	0.17083978
Adaptation	4	0.006885165	0.5025131	0.50251312
Partnership	4	0.007502492	0.4339186	0.15007837
Growth	5	0.00702816	0.3983348	0.39833478
Affection	**22**	**0.009057619**	**1.2877008**	**1.2877008**
Resolve	7	0.007565368	0.6963146	0.69631463
Hierarchical Plateau	11	0.006764162	**1.2538343**	**-0.49613444**
Content Plateau	8	0.00755348	1.0451572	**-0.42033123**
Centralization Plateau	5	0.006519002	0.7058435	0.05185634
Emotional Support	13	**0.00823591**	0.7653217	0.45627117
Instrumental Support	**20**	**0.008697011**	1.0365791	0.60325761
Goal Setting	6	0.006672364	0.9190835	**-0.6052166**
Continuous Learning	3	0.006992443	0.8300358	**0.83003579**
Self-Presentation	12	0.007664982	**1.0690933**	0.03692634
Relationship Building	**20**	0.007928738	0.9471995	**0.94719951**

Supplementary Table 2. CS Coefficients for Centrality Measures in the Network

Indicators	CS Coefficient
betweenness	0.205
closeness	0.205
expectedInfluence	0.750
strength	0.594
